# The Production of Albuminuria

**Published:** 1877-07

**Authors:** 


The Production of Albuminuria.—In an article in the *Transactions* of the Medical Society of King's county, N. Y:, Dr. W. H. Martin observes that the diseases which are now known to be attended by albuminuria are so numerous and pathologically so distinct, that we are puzzled in the endeavor to make analogy and comparison useful in testing the causative influence of pregnancy. It is hard to believe, for instance, that the conditions under which albuminuria is produced by valvular disease of the heart on one hand, and by diphtheria on the other, are identical, or even similar. That scarlatinal poison and that pregnancy both cause albuminuria is proved; but that both cause it by originating exactly the same kind of disturbance eludes demonstration. It is rather a "begging of the question" to assert that each produces changes in the blood, and that it is useless to seek beyond these wholly indeterminable changes for a mode of causation. It is easier to suppose that each disease or group of diseases (if they can be grouped etiologically or otherwise) has a peculiar power, and exerts it in a peculiar way, than it is to suppose the existence of some one essential condition to which all equally give rise; that is, one single and immediate cause of albuminuria.—*Med. and Surg. Reporter.*

